# Bioformulations with Beneficial Microbial Consortia, a Bioactive Compound and Plant Biopolymers Modulate Sweet Basil Productivity, Photosynthetic Activity and Metabolites

**DOI:** 10.3390/pathogens10070870

**Published:** 2021-07-10

**Authors:** Ernesto Comite, Christophe El-Nakhel, Youssef Rouphael, Valeria Ventorino, Olimpia Pepe, Assunta Borzacchiello, Francesco Vinale, Daniela Rigano, Alessia Staropoli, Matteo Lorito, Sheridan L. Woo

**Affiliations:** 1 Department of Agricultural Sciences, University of Naples Federico II, 80055 Portici, Italy; ernesto.comite@unina.it (E.C.); christophe.elnakhel@unina.it (C.E.-N.); youssef.rouphael@unina.it (Y.R.); valeria.ventorino@unina.it (V.V.); olipepe@unina.it (O.P.); alessia.staropoli@unina.it (A.S.); lorito@unina.it (M.L.); 2 Task Force on Microbiome Studies, University of Naples Federico II, 80055 Portici, Italy; 3 National Research Council, Institute for Composite Polymers and Biomaterials, 80125 Napoli, Italy; assunta.borzacchiello@cnr.it; 4 Department of Veterinary Medicine and Animal Production, University of Naples Federico II, 80137 Naples, Italy; frvinale@unina.it; 5 Institute for Sustainable Plant Protection, National Research Council, 80055 Portici, Italy; 6 Department of Pharmacy, University of Naples Federico II, 80131 Naples, Italy; drigano@unina.it

**Keywords:** 6-pentyl-α-pyrone, rosmarinic acid, *Ocimum basilicum* L., *Trichoderma*, *Azotobacter*

## Abstract

Increasing attention is being given to the development of innovative formulations to substitute the use of synthetic chemicals to improve agricultural production and resource use efficiency. Alternatives can include biological products containing beneficial microorganisms and bioactive metabolites able to inhibit plant pathogens, induce systemic resistance and promote plant growth. The efficacy of such bioformulations can be increased by the addition of polymers as adjuvants or carriers. *Trichoderma afroharzianum* T22, *Azotobacter chroococcum* 76A and 6-pentyl-α-pyrone (6PP; a *Trichoderma* secondary metabolite) were administrated singularly or in a consortium, with or without a carboxymethyl cellulose-based biopolymer (BP), and tested on sweet basil (*Ocimum basilicum* L.) grown in a protected greenhouse. The effect of the treatments on basil yield, photosynthetic activity and secondary metabolites production was assessed. Photosynthetic efficiency was augmented by the applications of the bioformulations. The applications to the rhizosphere with BP + 6PP and BP + T22 + 76A increased the total fresh weight of basil by 26.3% and 23.6%, respectively. Untargeted LC-MS qTOF analysis demonstrated that the plant metabolome was significantly modified by the treatments. Quantification of the profiles for the major phenolic acids indicated that the treatment with the T22 + 76A consortium increased rosmarinic acid content by 110%. The use of innovative bioformulations containing microbes, their metabolites and a biopolymer was found to modulate the cultivation of fresh basil by improving yield and quality, thus providing the opportunity to develop farming systems with minimal impact on the environmental footprint from the agricultural production process.

## 1. Introduction

Basil (*Ocimum basilicum* L.) is a member of the Lamiaceae family, which represents one of the most widely used medicinal and aromatic plants throughout the world [1,2]. There are many basil varieties, but the most commonly known cultivar is the sweet basil or Genovese basil, an important ingredient in the Mediterranean diet where it is used as a fresh leafy herb condiment or garnish, and is the major constituent in the “genovese” pesto sauce [3,4]. Members of the Lamiaceae, including basil, mint and salvia, are known to produce compounds such as phenolic acids and essential oils that provide the typical aromas attributed to the given plant species. Basil is known to contain antioxidant compounds [3,4] that can provide health benefits to consumers by protecting cells from damage evoked by oxidative stress and free radicals, accountable for numerous degenerative diseases [5,6]. Specifically, among the many secondary metabolites identified in basil, the essential oils are recognized as being effective in reducing antioxidant and antimicrobial stress [7], and the phenolic acids are known to have preventive protective effects on human well-being [5,6,8,9].

Phenolic compounds are secondary metabolites ubiquitous in plants that play an important role in chemical plant defense to pathogen-pest attack [10]. The presence of phenolic compounds in food or herbal products may be beneficial to human health upon regular consumption, since they may serve as functional food ingredients improving nutritional or nutraceutical properties and/or contributing to reduce some age-related diseases due to their antioxidant properties [10]. Among the numerous phenolic compounds present in basil, three phenolic acids are of particular interest due to their known applications in the field of human health: (i) *p*-coumaric acid and its conjugates are known for their antioxidant, anti-inflammatory, antitumor and anti-ulcer activities, playing an important role in mitigating arteriosclerosis, UV-induced eye damage, gout and diabetes [11]; (ii) caffeic acid has an inhibitory effect on the proliferation of tumor cells [12] and shows antioxidant activity both in vitro and in vivo [13]; (iii) rosmarinic acid, which is present in most plants of the Lamiaceae family [14], has antioxidant and pharmacological activities, as well as the ability to reduce allergies and pollinosis [15], plus it has demonstrated antimicrobial and insect-repellent capacities [16]. The accumulation and biosynthesis of phenolic compounds in basil have been noted to depend upon the plant genotype and physiology, plus the environmental factors, such as climate, cultivation technique and phenological phase of harvest [17]. In particular, the nutritional status of the crop farmed in a given agricultural system will have a direct impact on plant growth, subsequent metabolism and the produced phytochemical complex [4].

In recent years, there has been a growing interest among consumers and the scientific community in the search for innovative and eco-sustainable strategies to increase agricultural production, to meet food needs and reduce environmental impact. Among the possible solutions there is the use of plant biostimulants, agricultural products that include beneficial microorganisms (such as arbuscular mycorrhiza fungi and *Trichoderma* spp.) and natural substances (humic acids, seaweed and plant extracts, protein hydrolysate and silicon) able to stimulate plant vigor, growth and yield, even in sub-optimal conditions. These exemplify a valid alternative to chemical products that do not threaten biodiversity, able to reduce harmful effects to human health and the environment by decreasing the use of synthetic fertilizers and toxic pesticides [18,19,20,21]. The beneficial microorganisms that can be used as biostimulants include fungi such as *Trichoderma* spp. and bacteria belonging to *Azotobacter,* which can also be functionally complementary in a consortium acting as plant biostimulants [22]. Soil plant growth-promoting rhizobacteria (PGPR), such as the nitrogen-fixer *Azotobacter,* are important for their ability to produce regulatory and growth promoter compounds such as phytohormones, vitamins and antifungal metabolites, and to be involved in nutrient processes such as nitrogen cycling, phosphate solubilization [23], mobilization of iron [24] and the biodegradation of many commonly used pesticides, as demonstrated by *Azotobacter chroococcum* [25]. Other microorganisms with PGPR-similar effects include selected *Trichoderma* strains, capable of establishing diverse beneficial interactions with the plant, including biological control of pathogens, plant growth promotion (PGP) effects and induction of resistance [26,27,28]. *Trichoderma* spp. and other endophytic fungi have become more prominent on the agricultural scene in recent decades, owing to their beneficial effects and positive yield properties noted on crops [26,29]. Potentially new biological compounds to consider are secondary metabolites or bioactive substances from various microbial and plant sources that also have biostimulant or protective effects to the plant. *Trichoderma* spp. produce over 250 metabolic products, including secondary metabolites, peptides, proteins and cell-wall-degrading enzymes [22] with biological activity. For example, the 6 pentyl-α-pyrone (6PP), which produces the coconut aroma typical to some *Trichoderma* species, has demonstrated efficacy in the containment of known phytopathogenic fungi [30,31] and plant growth stimulation effects [32,33].

Consortia of beneficial microbes and bioactive compounds can be combined with natural and inorganic products such as algae, polymers and products of animal origin for more efficient and dependable agricultural formulations. Another innovative aspect, which responds to the current need for eco-sustainable products, comprises the use of macromolecules of natural origins, such as biopolymers. These substances can function as “carriers” of microbes (such as *Azotobacter* and *Trichoderma*) and/or PGP substances. The positive effects can be related to the in situ delivery and activity [34,35], as well as to the stabilization of microbial/natural compound formulations. Biopolymers can be formulated from biocompatible and biodegradable products, such as carbohydrate polymers, which have a great ability to absorb water and contain a large amount of nutrients and compounds of agricultural interest within their structure [36]. These polymers already find numerous useful applications in human health, including use as carriers in the delivery of some vaccines [37], anticancer drugs [38], antivirals [39] and therapeutic proteins and peptides [40]. Although, different studies investigated the role of some *Trichoderma* and PGPR strains in relation to their biostimulant action in horticulture [41,42], the combinatorial actions of beneficial microbial consortia and vegetal biopolymers have received very limited attention. Thus, exploiting the multiple properties of these beneficial microorganisms, in combination with a plant-based biopolymer, may represent promising strategies that target the formulation of more efficient biostimulant products.

Accordingly, the overall objective of this work was to evaluate bioformulations containing beneficial microorganisms, *Trichoderma afroharzianum* T22 (a fungus) and *Azotobacter chroococcum* 76A (a bacterium), the *Trichoderma* produced metabolite 6PP, applied singly or in consortia, with or without a biopolymer of plant origin in the cultivation of sweet basil, to determine effects on the plant (i) growth parameters, (ii) physiology, (iii) modulation of targeted and untargeted metabolites, and subsequently (iv) to identify the best bioformulation to enhance the desirable basil characteristics. The obtained results could be of major importance, contributing lines of research for developing new biological formulations for applications in agriculture, specifically to improve sweet basil production.

## 2. Results

### 2.1. Growth and Yield Parameters

The effects of the ten biological treatments on sweet basil were evaluated by measuring biometric parameters: leaf number per plant, harvested leaf yield fresh weight (FW), total aboveground plant biomass (leaves + stem) FW and dry weight (DW), root DW and percentage of total dry matter (DM) (Table 1). Water control (CTRL) plants registered the highest leaf number per plant, with BP + 6PP plants being similar, and the BP-treated plants had the lowest number of leaves. The paired applications of BP + 76A, BP + 6PP and T22 + 76A were similar among themselves, as were the single treatments with T22, 76A or 6PP. However, the highest leaf yield was noted in the plants treated with the combination of BP + T22 + 76A and the lowest with T22 + 76A. The total plant FW and DW were most positively influenced by the combinations of BP + 6PP or BP + T22 + 76A applied to the basil plants. The 6PP, BP + 6PP and BP + T22 + 76A treatments increased by 22.2% and 23.5% total FW and total DW on average, respectively, compared to CTRL treatment. Inoculation with 76A produced the greatest root DW, 52% higher than CTRL, whereas no significant differences were noted among the other formulations. It can also be mentioned that no disease symptoms were observed on the basil plants during cultivation in the field.

### 2.2. SPAD Index, Colorimetric Components

Both the SPAD index and the leaf colorimetric parameters of the basil plants were measured (Table 2). The bioformulations BP, T22, 76A, BP + T22, BP + 76A and T22 + 76A significantly increased the SPAD index of treated plants on average by 6.4%, in comparison to the control. As for the leaf colorimetric indices, none of the bioformulations had a significant impact on the brightness (L*), or b* indices. However, for the color parameter a*, BP + T22, 6PP and 76A applications showed significant differences in regard to the control (Table 2).

### 2.3. Physiological Parameters

Among the physiological parameters measured, only the net CO_2_ assimilation rate (A_CO2_) and the maximum quantum use efficiency of the photosystem II (Fv/Fm) demonstrated significant changes (*p* < 0.001) due to the bioformulation applications (Table 3). Stomatal resistance (r_s_) and transpiration rate E were not influenced by the different treatments. However, the CO_2_ net assimilation rate with the applications of BP, T22, 76A, BP + 76A and T22 + 76A bioformulations exhibited an increase of 11.8%, 11.8%, 16.0%, 9.3% and 11.3%, respectively, compared to CTRL. Among these latter bioformulations, except for T22 + 76A, all treatments had comparable Fv/Fm values that were higher than those recorded by CTRL. Furthermore, three of these treatments, with the single components, demonstrated Fv/Fm values higher than those recorded in the CTRL.

### 2.4. Untargeted Metabolomic Analysis and Compounds Differentially Expressed in the Organic Extracts

**Figure 1:**
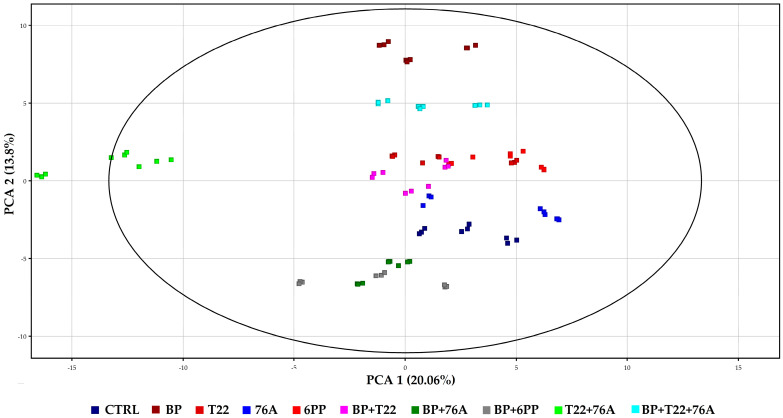
Analysis of the principal components (PCA) of the compounds identified in the leaf extracts of basil plants treated with the single components (BP, T22, 76A, 6PP, and CTRL) and with the relative combinations (BP + T22 + 76A, BP + 6PP, T22 + 76A, BP + 76A). The eigenvalues are represented with a total value of 33.86%, divided into PCA1 (20.06%) and PCA2 (13.8%).

**Figure 2:**
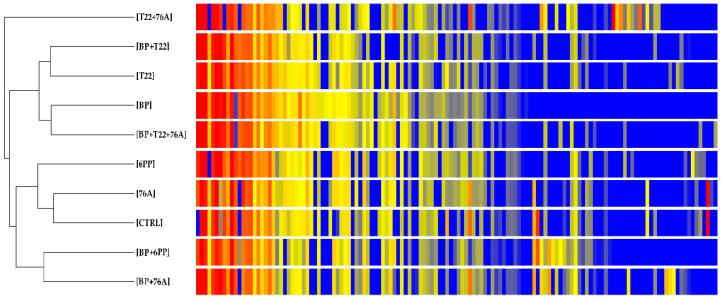
Abundance of the compounds in basil, treated with the bioformulations, as determined by LC-MS analysis and characterized in the heatmap. Plants were treated with bioformulations containing the single components (BP, T22, 76A, 6PP), the relative combinations (BP + T22 + 76A, BP + 6PP, T22 + 76A, BP + 76A, BP + T22), and a water (CTRL). The abundance of each compound is associated with a color scale ranging from blue (less abundant) to red (more abundant).

Untargeted LC-MS qTOF analysis of plant extracts indicated that the metabolomic profiles were significantly modified after the application of the bioformulates. In particular, multivariate analysis revealed the separation coupled to the chemical composition of the untargeted compounds of the treated basil, as clearly demonstrated in the Principal Component Analysis (PCA; Figure 1) and the hierarchical clustering (Figure 2). The untargeted LC-MS qTOF analysis of the basil leaf extracts allowed for the identification of 99 compounds that were differential in respect to the CTRL. Among these compounds, eighteen compounds were putatively identified by comparison to a database containing a collection of known characterized secondary metabolites (in house database). In particular, among the different phenolic compounds and flavonoids, it was noted the presence of three hydroxycinnamic acids, ferulic acid, *p*-coumaric acid, caffeic acid, as well as the caffeic acid ester rosmarinic acid (Table 4).

The PCA analysis (Figure 1) of all compounds indicated that the principal components (PCs) accounted for 33.86% of the total variance, PC1 20.06% and PC2 13.80%. As previously noted in the heatmap (Figure 2), the multivariate analysis clearly demonstrates the separation of treatment T22 + 76A from the other bioformulations in the ordination, positioned to the far left of PC1, whereas the other treatments grouped together in the center. The distribution along PC2 indicated a group of compounds determined by the presence of BP alone in the bioformulations, which were positioned in the upper quadrants of the graph, whereas, there were the groupings of the combinations, BP + 6PP and BP + 76A positioned at the bottom of the ordination. The hierarchical clustering analysis (Figure 2) highlighted a clear separation of the metabolic profile of the plants treated with T22 + 76A from the other treatments. The rest of the nine treatments grouped together were then separated into two groups, in which one cluster contained all T22 treatments, as well as the application of BP alone and the BP + T22 + 76A combination, whereas the second cluster contained 6PP, 76A and CTRL singly, plus the combinations with the biopolymer.

### 2.5. Targeted Metabolomic Analysis: Quantification of p-Coumaric, Caffeic and Rosmarinic Acids

**Figure 3:**
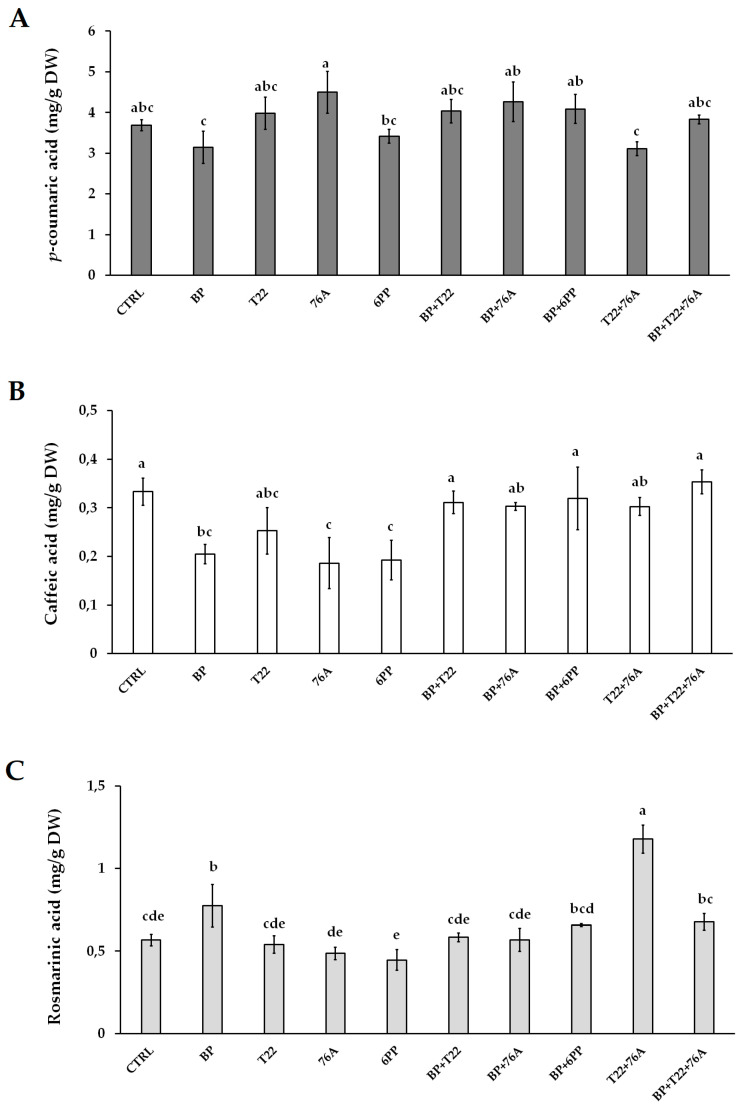
Concentration (mg/g DW) of *p*-coumaric acid (**A**), caffeic acid (**B**) and rosmarinic acid (**C**) in the leaf extracts of basil plants treated with the single components (BP, T22, 76A, 6PP), the relative combinations (BP + T22 + 76A, BP + 6PP, T22 + 76A, BP + 76A, BP + T22) and a water control (CTRL). Values are significant at *p* ≤ 0.001, and values followed by different letters are significantly different according to Duncan’s test at *p* ≤ 0.05.

The most typical and important phenolic metabolites in the basil extracts, *p*-coumaric, caffeic and rosmarinic acids, were quantified to commercial standards as a reference. In particular, the highest concentrations of *p*-coumaric acid were found in basil treated with 76A (4.49 mg/g DW), followed by BP + 76A and BP + 6PP; the lowest levels were noted in BP and T22 + 76A treatments (Figure 3A). The quantity of caffeic acid demonstrated that after treatments with BP + T22 + 76A, BP + 6PP and T22 + 76A, the values were similar to that of CTRL. For all other treatments, it can be noted that values were lower than CTRL, particularly for 6PP and 76A singular treatments, which registered the lowest values of caffeic acid, 0.19 and 0.18 mg/g DW, respectively (Figure 3B). For rosmarinic acid, the T22 + 76A treatment produced the highest concentration (1.17 mg/g DW) that was two-times higher than that of CTRL (0.56 mg/g DW). The lowest accumulation of rosmarinic acid was observed with the single treatments of 76A and 6PP, below the level of the CTRL (Figure 3C).

## 3. Discussion

Designing and formulating new microbial-based agricultural products, with biocontrol or biostimulant activity, is of particular interest for improving plant parameters such as growth promotion and yield, resistance to pathogens and pests, and the production of useful phytocompounds. This potential can be influenced by the individual microorganisms present in the bioformulations, and in some cases enhanced by the combined action of beneficial microbial consortia (i.e., containing endophytic fungi and/or PGPR), with their bioactive metabolites, and other natural components. In this study, the application of two beneficial microorganisms, *Trichoderma afroharzianum* T22, a fungal biocontrol agent, and *Azotobacter chroococcum* 76A, a nitrogen-fixing bacteria, a fungal secondary metabolite (6 pentyl-α-pyrone) and bio-based polymer were tested to determine the effects of the stand-alone or combined application on sweet basil. These microorganisms were able to colonize the soil rhizosphere, and in the plant–microbe interactions established, many physical and biochemical activities were stimulated that could increase plant/root system development as noted in other studies [26,33,41,42]. Furthermore, findings from other investigations were confirmed, indicating that this effect could be improved with the addition of macromolecules of natural origins, such as vegetal-based biopolymers [34,36,43]. In fact, the current work demonstrated that the microbial consortium with the BP induced a significant increase in yield fresh weight, total biomass and total dry weight when compared to the single microbial consortium and control treatments. The increased total fresh, dry biomass, and root dry weights observed in the present study were similarly noted by Sabra et al. [44] on basil treated with a combination of the beneficial fungi *Rhizophagus irregularis* and *Serendipita indica* in comparison to CTRL treatment. The growth-promoting action exerted by *Trichoderma*, causing a direct stimulation of root development, has been attributed to the release into the rhizo-soil of small peptides, auxins, volatiles and other active signaling compounds [26,27,31,41] or by the indirect manner through which the fungus influences the solubilization of soil minerals [26,45] to increase macro- and micronutrient availability, transport and/or plant absorption [29,33,41]. Many of these studies obtained similar results when plants (i.e., corn) were treated with the same *Trichoderma* beneficial fungus combined with a conventional fertilizer [26,41]. Moreover, Shirzadi et al. [46] obtained better agronomic traits in basil (plant height, shoot fresh weight and dry weight) when treatments included combinations of mycorrhizal fungi with *Azotobacter* that were associated to the secretion of molecules by the bacteria that affected plant growth, including vitamin B, nicotinic acid, gibberellin, cytokine, etc., other than its capacity for biological nitrogen fixation. In addition, basil plants inoculated with *Azotobacter* alone showed an increase in dry weight, as supported in a study by Roshanpour et al. [47].

Recently, Silletti et al. [48] reported the potential complementation of *Azotobacter* and *Trichoderma* as a PGP consortium, whereby the combined biological activities in the mixture were able to increase the plant biostimulation effect over that of the single component treatments. This could also be due to the multiple positive associations that occur among various microorganisms [49,50,51], which may provide both greater efficacy in disease control as well as plant growth promotion when compared to products containing the single microbial agent [52]. The present investigation indicated that the *Trichoderma* secondary metabolite (6PP), known for its auxin-like properties that effected plant growth [30] of different horticultural crops [32,33,53], was able to generate an increased plant growth-promoting effect on sweet basil when combined with the BP that was greater than that of the metabolite applied singly. The addition of the biopolymer to the microbes and the metabolite formulations provided a positive plant effect, possibly by improving the product composition and the mode of delivery of the active ingredients to the plant [34,35]. In addition, the carboxymethyl cellulose (CMC) composition of the biopolymer could also have provided a potential source of nutrients both to the microbial consortia of the bioformulation, the beneficial microorganisms in the rhizosphere, as well as to the plant itself, thus providing an overall improvement of plant fitness in these growth conditions [34,36,43,54]. The positive effect of the BP application alone was noted to improve the photosynthetic efficiency (Fv/Fm) of the basil in the current work, confirming the observations recently made by Carillo et al. [43], who observed that the single biopolymer treatment enhanced the production of some phytocompounds, such as GABA and MEA, involved in the photorespiration processes of tomato fruits.

The use of microorganisms in agriculture can also influence the rate and assimilatory pigments of photosynthesis; recent studies demonstrated the importance of microorganisms in physiological processes of plants. For example, fungi of the *Trichoderma* genus improved the chlorophyll synthesis of romaine lettuce and wall rocket plants [54,55], whereas *Azotobacter chroococcum* 76A increased the physiological parameters on microtome tomato [56]. In this study, all microbial treatments, particularly *Trichoderma afroharzianum* T22, showed higher chlorophyll values than the CTRL. In addition, the treatment with *Azotobacter chroococcum* 76A indicated greater phytostimulation efficacy with higher significant values of sweet basil in root dry weight, rate of CO_2_ net assimilation and photosynthetic efficiency that can be related to a better fitness of the plant. The colonization of roots by *Trichoderma* may enhance growth response due to the enhancement of carbohydrate metabolism, photosynthetic and respiratory rates [45], thus triggering the plants physiological processes that improve the photosynthesis rate and stomatal conductance [57]. Silletti et al. noted that the photosynthetic rate was similarly higher in wheat when treated with *Trichoderma* or with *Azotobacter* [48], whereas the bacteria was found to release phytohormones that could stimulate photosynthesis in basil [47]. The co-inoculation of *Piriformospora indica* and *Trichoderma virens* was found to produce an improvement of chlorophyll fluorescence parameters [58], whereas, in the present study, only the inoculation of T22 alone demonstrated an improvement in photosystem II efficiency in basil. In addition, Gonzalez-Rodriguez and co-workers [59] depicted an increase in the photosynthesis parameters, such as total chlorophyll and photosynthesis in pineapple in vitro plantlets treated with *Azotobacter chroococcum*.

On the global market, much attention is being given by consumers to purchase foods that have been cultivated in low-environmental-impact systems, i.e., organic farming, or with reduced chemical products (fertilizers and pesticides), and to select nutrient-dense products that have a higher health value and nutritional content. This perspective has fostered a growing interest in the development of research and cultivation practices focused on improving the properties of the plant compounds found in various food products in order to provide an essential human diet that contributes to the overall well-being of the consumer.

Basil has been widely used in traditional medicine [60,61] as a digestive stimulant [62] and is recognized for its antibacterial [63], antitumor [60] and anticonvulsant [64] properties. In particular, basil is rich in phenolic acids, which contribute to its strong antioxidant capacity [65,66,67], a property that exerts beneficial effects on human health—the vascular and nervous system [8]—reducing the effects associated with various degenerative diseases such as Alzheimer’s [5], Parkinson’s [6] and dementia [9]. These compounds are known to positively protect key biological constituents such as lipoproteins, membranes and DNA from oxidative processes [68]. Due to the importance of phenolic compounds for consumer health, their quantity indirectly attributes an extra value to the crops that improves the nutritional and functional properties of vegetables and herbs [69].

The evaluation of the phenolic components produced by the plant in response to the application of diverse bioformulations during cultivation in the field indicated that the metabolic profile of the different basil leaf extracts was highlighted by a differential abundance of biologically important metabolites, including p-coumaric acid, caffeic acid, and rosmarinic acid, known for their health properties [11,12,13,15,16]. In particular, this study shows that the treatment of basil plants with *Azotobacter chroococcum* 76A alone increased the production of p-coumaric acid, while the *Trichoderma afroharzianum* T22 + *Azotobacter chroococcum* 76A treatment induced a major production of rosmarinic acid. In general, the quantity of caffeic acid in the basil extracts was less affected by the biological treatments, and not clearly associated to specific treatments.

Other studies have demonstrated how the use of microorganisms can influence the essential oil and phenolic components on basil. For instance, the inoculum of AMF improved the concentration of rosmarinic acid, chicoric acid and caffeic acid on 4 basil cultivars [70], growth and aroma volatiles (e.g., linalool) on sweet basil in heavy-metal-contaminated soil [44], plus the use of different commercial microbial bio-based products increased the percentage of different metabolites (e.g., caffeic acid) on different basil cultivars [57]. Moreover, our results indicate that the simultaneous application of *Trichoderma afroharzianum* T22 with *Azotobacter chroococcum* 76A induced a better modulation of phenolics metabolism when compared to the single applications of *Trichoderma* and *Azotobacter*, especially for rosmarinic and caffeic acid, as is in line with findings by Sabra et al. [44] affirming that the dual application of beneficial fungi and its associated bacteria generated an enhancement of sweet basil nutraceutical value.

The application of the microbial, fungal bioactive compounds and biopolymer components in various bioformulations was found to differentially modify the agronomic characteristics and the metabolic profile of basil plants, in some cases increasing the quantity of the phenolic compounds, thus producing a qualitatively superior final product. In fact, phenolics strongly contribute to basil antioxidant capacity and biological properties [65,66,67], so through the increase of these components, well known for their many applications in the field of human health [11,12,13,15,16], the consumer can obtain a more valuable basil with enhancing health properties. However, ongoing studies will determine the effects of these biological formulations on Genovese basil and the other phytocompounds it produces, such as essential oils, that are important for the characteristics of aroma and the biocontrol of disease agents, that contribute to the unique qualities of this Mediterranean food and medicinal plant.

## 4. Materials and Methods

### 4.1. Application of Microbial Biostimulants

The microbial biostimulant treatments involved the use of different microorganisms, their components and natural molecules. Nine biostimulant treatments were adopted: (i) *Trichoderma afroharzianum* strain T22 (ex-*Trichoderma harzianum* [71]) commercial formulation of Trianum-P (Koppert Biological Systems, Rotterdam, the Netherlands) implemented at a final concentration of 10^7^ spore mL^−1^, (ii) *Azotobacter chroococcum* strain 76A ([72]; freeze-dried bacterial cells in a final concentration of 10^7^ CFU mL^−1^), (iii) 6 pentyl-α-pyrone (6PP) concentration of 10^−6^ M (Sigma-Aldrich, Milan, Italy), (iv) biopolymer (BP; diluted in water) composed of carboxymethyl cellulose (CMC), a polyanion derived from cellulose used as a thickener, emulsifier and nutrient carrier in agriculture with the addition of Pluronic F-127 (PF-127), as reported in [43], (v) T22 (10^6^ spores mL^−1^) + 76A (10^7^ CFU mL^−1^), (vi) BP + T22 (10^7^ spores mL^−1^), (vii) BP + 76A (10^7^ CFU mL^−1^), (viii) BP + T22 (10^6^ spores mL^−1^) + 76A (10^7^ CFU mL^−1^) and (ix) BP + 6PP (10^−6^ M); additionally, (x), a control treatment, only water, was administrated at the same volume as the other treatments. The liquid bioformulations with the microorganisms and the metabolite were prepared in water throughout the experiment. The treatments containing the biopolymer were always diluted with water in a 1:1 proportion (BP: water), which included the final concentration of the other components in the total final volume of the bioformulation.

### 4.2. Plant Material, Greenhouse Experimental Design and Treatments

Sweet basil (*Ocimum basilicum* L. cv Genovese) was used in this experiment, carried out in a protected greenhouse of the BiPaF Section of the Department of Agriculture, University of Naples Federico II, Portici (NA, Italy). A randomized complete-block scheme with a total of 10 treatments was replicated three-times (total 300 plants). Commercial seedlings were transplanted to the field in June. The first application of the biological treatments was carried out at the time of transplant using a root dip method [43,55]. After two weeks, the treatments were repeated by watering 25 mL of the bioformulation treatments to the base of each plant. Plants were observed weekly for any developing leaf or root disease symptoms.

### 4.3. Sampling and Yield Assessment

In July 2019, 34 days after transplanting, the plants were cut above the first node. For each treatment, 5 plants were harvested per replicate, for a total of 15 plants per treatment, from which destructive biometric analyses were conducted to evaluate the marketable production. In particular, the number of leaves, yield fresh weight (FW of the leaves) and total biomass (leaves + stems) FW were measured immediately after harvest. Subsequently, a sub-sample of each plant fresh sample, plus the washed roots, were placed in a forced-air oven at 65 °C for approx. 72 h, to obtain a constant dry weight (DW) of the plant material, then total dry matter (DM%) was calculated as DW/FW × 100.

### 4.4. SPAD Index and Colorimetric Components

SPAD index measurements were performed on young fully expanded basil leaves (between midrib and leaf margin) using a Chlorophyll Meter (Minolta SPAD-502, Osaka, Japan). A total of 30 measurements were acquired per replicate and reported to one mean value. Leaf colorimetry, was determined by measuring the colorimetric indices (L*, a*, b*) of 10 young fully expanded leaves per replicate using a colorimeter Minolta Chroma meter, CM-2600d (Minolta Camera Co. Ltd., Osaka, Japan).

### 4.5. Determination of Leaf Gas Exchange and Photosystem II Efficiency

Before harvest, a portable gas exchange analyzer (LCA-4; ADC BioScientific Ltd., Hoddesdon, UK) was used to determine the net assimilation rate of CO_2_, the stomatal resistance and the transpiration rate of basil plants (A_CO2_, r_s_ and E, respectively). Three physiological measurements were determined per replicate. For the maximum quantum use efficiency of the Photosystem II (Fv/Fm), measurements were performed with a portable fluorometer (Plant Stress Kit, Opti-Sciences, Hudson, NH, USA), where four measurements per replicate were performed.

### 4.6. Preparation of Basil Leaves Extracts

For the metabolomic analysis, fresh leaf samples were collected from each replicate, submerged in liquid nitrogen, then stored at −80 °C. The samples were freeze dried for 72 h, and the leaf tissue was pulverized using a mortar and pestle. A 200 mg aliquot of the lyophilized powdered material was suspended in 2 mL of an 80:20 methanol/H_2_O solution (solvent 99.9% for LC-MS). The sample was agitated for 1 min by vortexing, then centrifuged for 15 min at a speed of 5000 rpm at a temperature of 4 °C. The supernatant was filter sterilized (0.22 µm syringe filter) and stored in glass vials, at 4 °C until analysis.

### 4.7. LC-MS analysis—Targeted and Untargeted Metabolome

Following the method described by Marra et al. [73], the spectrometric analysis of the plant extracts was performed by an LC-MS Q-TOF Agilent Technologies (Santa Clara, CA, USA), equipped with a 1260 Infinity series HPLC with DAD detector, and a mass spectrometer Q-TOF (model G6540) with Dual ESI source. The plant extracts were separated with a reverse-phase analytical Ascentis ^®^ Express C18 column (2.7 μm, 50 mm × 3.0 mm id, Supelco ©, Bellefonte, PA, USA). The identification and quantification of the three phenolic acids *p*-coumaric, caffeic and rosmarinic were obtained by comparing the mass and the retention time (RT) to standard compounds (Sigma-Aldrich, St. Louis, MO, USA). These standards were analyzed with the same LC-MS method and the quantification was obtained by interpolating the averaged data with a previously constructed calibration curve.

### 4.8. Statistical Analysis

Data were statistically analyzed (One-way Anova) with SPSS v. 21 (IBM Corp., Armonk, NY, USA). Bioformulations of beneficial microbial consortia and vegetal biopolymers effects on yield, growth parameters, SPAD index, leaf colorimetry, physiological analysis and phenolics profile were analyzed using Duncan’s multiple range test performed at *p* = 0.05. Statistical analysis of basil extracts metabolic profile was carried out using Mass Profile Professional, version 13.1.1 (Agilent Technologies). One-way ANOVA (*p* > 0.05) with Tukey–HSD post hoc was implemented to evaluate the differential significance of samples. Eventually, a fold change > 2.0 was used. The results obtained were then subjected to principal component analysis (PCA) to depict the difference between the different biostimulant treatments. A grouping of the samples was then made based on the abundance of continuous variables (Hierarchical clustering) by combining the technical replicates. Statistically relevant compounds were identified using: an in-house database containing information on over 2000 secondary metabolites produced by plants; the library METLIN provided from Agilent, containing over 15,000 natural metabolites and di- and tri-peptides; information available from literature.

## 5. Conclusions

The implementation of bioformulations with the association of specially selected microorganisms, bioactive substances and biopolymer allowed us to define and present new bioformulations for use in agriculture. The outcome of the developed bioformulations consisted of improved parameters of Genovese basil, in terms of growth promotion, yield increase and the efficiency of photosystem II and photosynthesis in general. Furthermore, these treatments significantly modulate the plant metabolome and increase differentially the production of three beneficial phenolic compounds: *p*-coumaric acid, caffeic acid and rosmarinic acid. In summary, for the agronomic parameters evaluated, the highest fresh weight yields (marketable produce) were noted in the plants treated with the combinations of BP + T22 + 76A or BP + 6PP. Regarding the biochemical profiles of the phenolic plant compounds in basil, plants receiving the T22 + 76A treatment exhibited the greatest production of rosmarinic acid, whereas *Azotobacter chroococcum* 76A alone displayed the best increased production of *p*-coumaric acid. Therefore, the new formulations based on a consortium of *Trichoderma*, *Azotobacter*, 6 pentyl-α-pyrone and a plant biopolymer are presented as innovative products in improving the production of Genovese basil. The application of these bioformulations can have a dual positive effect, achieving both better eco-sustainable agriculture and the opportunity to obtain a final product with improved yield and bioactive secondary metabolites content.

## Figures and Tables

**Table 1:** Effect on the biometric parameters of basil plants treated with the different bioformulations containing the biopolymer (BP), *Trichoderma afroharzianum* (T22), *Azotobacter chroococcum* (76A), or the secondary metabolite (6PP), plus a water control (CTRL), used individually or in combination.

Treatment	Leaf Number	Leaf Yield FW	Aboveground Biomass FW	Aboveground Biomass DW	Root DW	DM
	(No. plant^−1^)	(g plant^−1^)	(g plant^−1^)	(g plant^−1^)	(g plant^−1^)	(%)
CTRL	290.6 a	93.85 bcd	153.0 cd	17.48 c	4.44 d	11.42
BP	208.7 f	92.81 bcd	166.8 bc	18.58 bc	4.79 cd	11.14
T22	236.6 cde	86.52 cd	151.2 cd	17.54 c	4.99 bcd	11.59
76A	219.8 ef	94.15 bc	159.7 bc	18.87 bc	6.75 a	11.80
6PP	226.1 def	90.83 bcd	178.3 ab	20.95 ab	5.24 bcd	11.76
BP + T22	227.4 def	86.64 cd	148.2 cd	16.54 c	5.81 b	11.18
BP + 76A	255.7 bc	87.06 cd	153.0 cd	17.71 c	5.59 bc	11.61
BP + 6PP	272.8 ab	106.26 ab	193.2 a	21.56 a	4.72 cd	11.16
T22 + 76A	265.2 b	77.41 d	130.8 d	16.19 c	5.17 bcd	12.39
BP + T22 + 76A	241.5 cd	109.62 a	189.1 a	22.19 a	5.67 bc	11.73
Significance	***	**	***	***	***	ns

Different letters within each column indicate significant differences according to Duncan’s multiple-range test (*p* = 0.05). ns, **, *** non-significant or significant at *p* ≤ 0.01 and 0.001, respectively.

**Table 2:** Effect of the different bioformulations with the biopolymer (BP), *Trichoderma afroharzianum* (T22), *Azotobacter chroococcum* (76A), the secondary metabolite (6PP) and water (CTRL), used individually or in combination, on SPAD index and leaf colorimetric indices (L*, a* [−a* = green)], b* [(+b* = yellow]) of basil plants.

Treatment	SPAD Index	L*	a*	b*
CTRL	33.95 d	41.69	−6.80 c	14.65
BP	36.17 ab	41.10	−6.30 abc	13.38
T22	36.97 a	41.17	−6.46 bc	14.05
76A	35.78 ab	41.75	−6.22 ab	13.47
6PP	34.26 cd	41.24	−6.20 ab	12.96
BP + T22	35.30 bc	41.09	−5.87 a	12.45
BP + 76A	36.19 ab	42.03	−6.37 abc	13.14
BP + 6PP	33.80 d	41.78	−6.52 bc	14.48
T22 + 76A	36.28 ab	41.78	−6.27 abc	13.08
BP + T22 + 76A	35.09 bcd	40.05	−6.61 bc	13.87
Significance	***	ns	*	ns

Different letters within each column indicate significant differences according to Duncan’s multiple-range test (*p* = 0.05). ns, *, *** non-significant or significant at *p* ≤ 0.05 and 0.001, respectively.

**Table 3:** Effect on the physiological parameters of basil plants: rate of CO_2_ net assimilation (A_CO2_), stomatal resistance (r_s_), transpiration rate (E) and photosystem II efficiency (Fv/Fm) of the different formulations with the biopolymer (BP), *Trichoderma afroharzianum* (T22), *Azotobacter chroococcum* (76A), or the secondary metabolite (6PP), and a water control (CTRL), used individually or in combination.

Treatment	A_CO2_	r_s_	E	Fv/Fm
	(μmol CO_2_ m^−2^ s^−1^)	(m^2^ s^1^ mol^−1^)	(mol H_2_O m^−2^ s^−1^)	
CTRL	15.75 d	3.85	4.62	0.81 bc
BP	17.61 ab	4.69	4.27	0.82 a
T22	17.62 ab	4.90	4.48	0.83 a
76A	18.28 a	4.05	4.46	0.82 a
6PP	16.55 cd	4.77	4.24	0.82 ab
BP + T22	16.43 cd	3.84	4.80	0.81 bc
BP + 76A	17.22 bc	4.43	4.52	0.81 bc
BP + 6PP	16.48 cd	5.18	4.41	0.79 d
T22 + 76A	17.54 ab	4.36	4.86	0.80 c
BP + T22 + 76A	16.55 cd	4.59	4.49	0.81 c
Significance	***	ns	ns	***

Different letters within each column indicate significant differences according to Duncan’s multiple-range test (*p* = 0.05). ns, *** non-significant or significant at *p* ≤ 0.001, respectively.

**Table 4:** Metabolites putatively identified in leaf extracts of basil plants treated with different bioformulations in the field. For each compound, mass, retention time (RT), chemical empirical formula and similarity score are reported.

Compound	Mass	RT	Chemical Empirical Formula	Similarity Score
Isocitric acid	192.0279	0.976	C_6_H_8_O_7_	83.63
Caffeic acid	180.0418	2.362	C_9_H_8_O_4_	87.15
4-hydroxybenzoic acid	138.0319	2.853	C_7_H_6_O_3_	85.75
Luteolin-3-*O*-glucuronide	448.1219	3.888	C_21_H_18_O_12_	98.66
Ferulic acid	194.0577	4.159	C_10_H_10_O_4_	86.95
Lupinisoflavone E	438.1652	4.182	C_25_H_26_O_7_	67.85
Phenylacetic acid	136.0518	4.336	C_8_H_8_O_2_	86.73
Tricetin 3′-methyl ether 7-glucuronide	492.0885	4.439	C_22_H_20_O_13_	87.54
Medioresinol	388.1731	4.463	C_21_H_24_O_7_	97.86
Foliasalacioside A2	434.2136	4.644	C_19_H_32_O_8_	93.11
Apigenin-7-*O*-glucoside	432.1993	4.714	C_21_H_20_O_10_	83.72
7-hydroxycoumarin	162.0316	5.006	C_9_H_6_O_3_	86.6
Rosmarinic acid	360.0843	5.015	C_18_H_16_O_8_	99.32
Quercetin-5,3′-dimethyl ether-3-glucoside	492.1269	5.026	C_23_H_24_O_12_	49.67
*p*-coumaric acid	164.0837	5.148	C_9_H_8_O_3_	87.36
Cirsimaritin	314.079	5.613	C_17_H_14_O_6_	99.65
Rotundic acid	488.3499	6.571	C_30_H_48_O_5_	96.79
Colneleic acid	294.2194	6.961	C_18_H_30_O_3_	98.47
